# β-Carbolines in Experiments on Laboratory Animals

**DOI:** 10.3390/ijms21155245

**Published:** 2020-07-24

**Authors:** Renata Zawirska-Wojtasiak, Agnieszka Fedoruk-Wyszomirska, Paulina Piechowska, Sylwia Mildner-Szkudlarz, Joanna Bajerska, Elżbieta Wojtowicz, Krzysztof Przygoński, Dorota Gurda, Wiktoria Kubicka, Eliza Wyszko

**Affiliations:** 1 Faculty of Food Science and Nutrition, Poznań University of Life Sciences, Wojska Polskiego 28, 60-637 Poznań, Poland; paulina.piechowska@up.poznan.pl (P.P.); sylwia.mildner-szkudlarz@up.poznan.pl (S.M.-S.); joanna.bajerska@up.poznan.pl (J.B.); 2 Institute of Bioorganic Chemistry, Polish Academy of Sciences, Noskowskiego 12/14, 61-794 Poznań, Poland; agaw@ibch.poznan.pl (A.F.-W.); d_gurda@ibch.poznan.pl (D.G.); wiktoriakubicka.k@gmail.com (W.K.); 3 Department of Food Concentrates and Starch Products, Prof. Wacław Dąbrowski Institute of Agricultural and Food Biotechnology, Starołęcka 40, 61-361 Poznań, Poland; ewojt@man.poznan.pl (E.W.); krisp@man.poznan.pl (K.P.)

**Keywords:** β-carbolines, chicory, rats, behavioral tests, in vitro experiments

## Abstract

Some studies have ascribed a protective effect against neurodegenerative diseases to the β-carbolines harman (H) and norharman (NH), which occur mostly in coffee and coffee substitutes. We determined the concentrations of β-carbolines and undesirable compounds (such as acrylamide) in roasted coffee substitute ingredients and found that chicory coffee was optimal. Two in vivo experiments were conducted with seventeen-month-old male Sprague Dawley rats fed a diet with the addition of pure carboline standards in the first stage, and chicory in the second. We observed an increase in the level of H and NH in blood plasma, as well as higher activity of animals in the battery behavioral test, particularly in the second stage. The results of in vitro studies—particularly the level of the expression in brain tissue of genes associated with aging processes and neurodegenerative diseases—clearly show the benefits of a diet rich in β-carbolines.

## 1. Introduction

β-carbolines are biologically active, naturally occurring plant-derived alkaloids that are derivatives of indole. Norharman (NH) and harman (H) are the most frequently identified β-carbolines. The literature has reported the presence of NH and H in processed and stored food [1,2]. In recent years, attention has turned to the neuroactive effects of NH and H [3,4]. Carbolines can act as neuromodulators through their effect on monoamine oxidase (MAO), which may lead to depression, Parkinson’s disease (PD), and Alzheimer’s disease [4]. However, some studies on carbolines have suggested that there may be specific MAO inhibitors. Research on the expression of this enzyme focuses on effectively inhibiting its action, which would allow treatment of both affective disorders and Parkinson’s disease [5,6]. Monoamine oxidase inhibitors were among the first antidepressants to be discovered [5]. Studies on the activity of MAO in tobacco point to the high level of enzyme inhibitors in the form of NH and H in the brain of smokers [2]. It was also shown by other authors [7] that a potent monoamine oxidase inhibitor and stimulant is H, while NH has a calming effect on the body. There have been few animal experiments performed with these compounds and diseases [3,8]. Some studies have investigated their antidepressant action on mice, showing that positive effects were associated with the addition of β-carbolines to experimental feed (H and NH, in doses of 5–15 mg/kg i.p. and 2.5–10 mg/kg i.p., respectively) [8]. Positive effects were also observed by Celikyurt et al. [3] in rats, where H was administered in doses of 2.5 mg/kg, 5.0 mg/kg, and 7.5 mg/kg, with the two higher doses leading to significantly fewer errors in working memory.

Li et al. [9] examined the bioavailability of β-carbolines on living organisms, using harman at 1 mg/kg and 30 mg/kg body weight; they calculated its bioavailability as 19.41% via oral administration.

Experimentation on animals (usually mice, hamsters, and rats) allows the actions and effects of compounds to be assessed. Carbolines added to fodder or intraperitoneally have been measured in the blood and organ before and after death [3,10]. There have been studies of the supplementation of fodder with natural sources of carbolines, such as coffee substitute. Some compounds play a neuroprotective role through the synthesis of active molecules, activation of appropriate receptors, or exertion of an effect on enzymatic changes, thus preventing the degradation of neurons and inhibiting the massive loss of important cells. The main obstacle, as for medicines, is likely to be the blood–brain barrier, which protects the brain against external factors while limiting the absorption of drugs [11].

An alternative to animal experiments in studying this phenomenon is represented by modern in vitro experiments on cell lines; however, according to Martinez-Morales and Liste [12], progress here can be difficult because of the lack of predictive and other cellular models. Nonetheless, some significant observations have been made in relation to Parkinson’s disease in such studies.

Our literature review strongly suggests that some foods, especially coffee, can act as a rich source of β-carbolines, which may be associated with a reduced risk of serious neurodegenerative diseases, such as Parkinson’s and Alzheimer’s [13]. Human cancer cell lines have also been used in studies on the antitumor effects of some carboline derivatives [14].

The aim of our study is to use an animal experiment to determine the neuroactive effect of β-carbolines supplied as pure compounds and in a natural source (coffee substitute). We examined the influence of β-carbolines on the condition of aged rats by behavioral tests and analysis of the expression level of genes associated with neurodegenerative changes, aging, and apoptosis in the brain tissue of treated animals. Moreover, to determine the biological effect of analyzed β-carbolines on aged human cells we investigate cytotoxicity, reactive oxygen species (ROS) activation, and cell cycle changes using two models of cell lines: MRC-5 derived from a male fetus and AG20445A from an old patient diagnosed with PD. Both cell models were suitable for further aging needed to conduct our research.

The research hypothesis is that adding β-carbolines to the feed in the form of a coffee substitute protects experimental animals from neurodisorders.

## 2. Materials and Methods

### 2.1. Chemical Standards 

Chemical standards of harman and norharman were sourced from Fluka, Steinheim, Germany and Sigma-Aldrich, Steinheim, Germany. Other chemicals came from Sigma-Aldrich or AlfaChem, Poznań, Poland.

### 2.2. Raw Materials

Dried chicory (Cichorium intybus) was obtained from the production plant of Cykoria, Wierzchosławice, Poland.

### 2.3. The Experimental Diet 

The experimental diet was prepared by ZooLab (Urszula Borgiasz, Sędziszów, Poland) from corn starch 465.69 g/kg, casein (>85% of proteins) 140.00 g/kg, maltodextrins 155.00 g/kg, sucrose 100.00 g/kg, soya oil (without additives) 40.00 g/kg, fiber (α-cellulose), 50.00 g/kg, mineral mix 35.00 g/kg, vitamin mix 10.00 g/kg, l-cystine 1.80 g/kg, choline 2.50 g/kg, and tert-butylhydroquinone 0.008 g/kg enriched with carbolines (for stage 1 of the experiment), in concentrations equivalent to the contents of these compounds found in coffee consumed by humans in a volume of 0, 500, 750, and 1000 mL daily (H1: 10 µg/kg animal diet; H2: 15 µg/kg animal diet; H3: 20 µg/kg animal diet; NH1: 8 µg/kg animal diet; NH2: 12 µg/kg animal diet; NH3: 16 µg/kg animal diet) and with chicory at 12.5 g/kg animal diet (stage 2).

### 2.4. Animal Experiment

The experiment was conducted in the animal housing facility at the Institute of Human Nutrition and Dietetics, Poznań University of Life Sciences, under conditions of natural light and with the temperature maintained during the experiment at 19–22 °C, and with a relative humidity of 55–60%. Seventeen-month-old male Sprague Dawley rats originating from a Charles River Laboratories farm were used in the experiment. The age of the rats corresponded to 51 years of human life [15]. For the first ten days of the experiment (the adaptation period), the rats were fed ad libitum with a standard laboratory diet and water. The experiment was conducted in two stages. In the first stage, after acclimatization, the animals were randomly divided into seven groups (*n* = 6 each). The control rats were fed the AIN-93M rodent diet. The experimental rats were fed the same diet enriched with harman or norharman standards at a concentration equivalent to its concentration in coffee consumed by humans in amounts of 500, 750, and 1000 mL daily. In the second stage of the study, after an acclimatization period, the animals were randomly divided into two groups (*n* = 6 each) and fed either the AIN-93M (control) diet or the same diet enriched with dried chicory, as a natural source of β-carbolines, in the amounts indicated during the first stage as being most effective for improving cognitive functions in the animals. There were no differences in body weight between the groups at the beginning of either stage of the experiment. All rats were provided ad libitum diet and water for the fourteen weeks of both experiments. The food consumption of the individual rats was monitored daily by measuring the difference between the amount of diet supplied each day and the amount of diet remaining. Feces were collected for ten days in the seventh week of the experiment, and water content, protein, fat, dietary fiber, and ash were determined using standard analytical methods. Body mass was measured every seven days using an electronic balance. At the end of the experiment, animals were subjected to a series of behavioral tests (classical labyrinth test) and the forced swim test (FST). Additionally, the animals in stage 2 of the experiment were subjected to the open field test (OFT). At the end of the study, and following 16 h of starvation, the animals were weighed and euthanized by a mixture of air and CO_2_ at a concentration of 30%/70% using an Easy-Box Euthanasia System (Animalab, Poznań, Poland). They were then dissected to collect blood from the hearts for future biochemical analysis of β-carboline levels.

The animal study design was approved by the local animal studies bioethics committee in Poznań, Poland (approvals 51/2017 and 29/2019) and performed according to the European Communities Council Directive of 24 November 1986 (86/609/EEC).

### 2.5. β-Carboline Determination in Feed, Chicory, and Animal Blood

The β-carboline levels in chicory were measured following Wojtowicz, Zawirska-Wojtasiak, Przygoński, and Mildner-Szkudlarz [16], with some modifications of sample dose (1–4 g) and validation (repeatability of H and NH 8.9% and 5.8%, recovery of H and NH 100.2% and 96.4%). The extracts were separated on a Dionex LC HPLC system with a Supelco C18 column and an RF-2000 detector.

The β-carboline levels in the experimental diet were determined based on the method described by Adachi et al. [17], with appropriately selected process parameters. To increase the accuracy of the process, double cold extraction was used to extract the test compounds from the experimental feed. Hot extraction was not employed due to the danger of pasting. Seven trials were made.

Determining the β-carbolines in blood required special preparation of the samples, following Zheng, Wang, Barnes, Guan, and Louis [18]. The heparin and plasma collected in the course of the section were placed in liquid nitrogen and stored at −20 °C. Immediately prior to extraction, the samples were brought to room temperature. For the analysis, samples of approximately 5–10 mL were used in duplicate from each individual. Initially, a sample of blood was placed in Falcon conical tubes (50 mL capacity) and mixed with half the volume of 1.0 M NaOH (2:1). The mixture was manually shaken at room temperature for about 1–2 min, and then on a horizontal shaker for 30 min, to break down blood cells and solubilize protein components. After shaking, the extraction solution was added to the samples in a volume equal to that of the mixture in the test tubes. The extraction solution consisted of ethyl acetate and methyl t-butyl ether in a ratio of 2:98. The extraction was carried out using a horizontal shaker at room temperature for 45 min. After extraction, the mixture was centrifuged at 3000× *g* for 10 min. The upper organic phase was removed and transferred to a new Falcon conical tube. Extraction on the sample was carried out an additional two times, and the organic phase was stored in a refrigerator until the extracts had combined. The combined extracts were then evaporated under dry nitrogen. The dry residue was dissolved in 0.5 mL of methanol. The samples prepared in this way were stored at −19 °C.

The concentration of H and NH in these samples was then estimated by HPLC. A Dionex Instrument with a P680 pump, ASI-100 autosampler, TCC-100 thermostat, and RF-2000 fluorescence detector was used. Separation was performed on Thermo Science Hypersil C18 column (3 mm × 150.3 µm), heated to 30 °C; the injection volume was 50 µL. The mobile phase consisted of deionized water containing 0.03 M sodium formate and 0.025 M triafluoroacetic acid in 1 dm^3^ (A), and acetonitrile (B).

Gradient: 0 min 0% B to 5 min 20% B, 14 min 20% B, 14.5 min 0% B, 19 min 0% B. Fluorescence excitation was induced using a 300 nm wavelength, and detection was performed at 440 nm. Peaks were identified by the expected retention times for H and NH standards. The calibration curve was determined based on the prepared standard solutions with known concentrations of β-carbolines ranging from 0 to 20 ng/mL. The correlation coefficients were 0.9979 for H and 0.9983 for NH. The recovery rates for harman and norharman were respectively 72.1% and 21.3%. The results for H and NH were corrected for the designated recovery.

### 2.6. Behavioral Tests

#### 2.6.1. Classical Labyrinth Test

A classic labyrinth (110 × 65 cm) was used, with corridors about 15 ± 15 cm, located on the table raised 50 cm from the ground. The test was performed in an isolated behavioral testing room with a low-intensity white light source. The time needed to complete the route between the two points of departure and arrival was measured. After each trial, any urine and fecal matter was removed, and the labyrinth was cleaned.

#### 2.6.2. Open Field Test (OFT)

The test was performed in a rectangular cage (80 × 40 cm) with dark walls and a white floor. The animal was placed in the cage arena for five minutes and its behavior was recorded for later analysis, as mobility time, time spent in cage center, and self-grooming frequency. The OFT was always performed at the same time of day, and the cage was cleaned between each test [1].

#### 2.6.3. The Forced Swim Test (FST)

The forced swim test was performed as described by Porsolt, Pichon, and Jalfre [19]. The experiment was performed in two sessions in an isolated behavioral testing room. In the first session, the pretest, animals were forced to swim for 15 min. The second session, which was the actual test, was conducted 24 h after the pretest for 5 min only. A transparent cylinder with water at 23–25 °C was used, with the water being freshly changed for every rat. After each test session, the animals were dried briefly with a towel and returned to their home cages. The animals’ behavior was recorded using a video camera and scored manually. Rats were described as immobile if they performed no activity other than that required to maintain the head above the water. In addition to immobility time, episodes of head shakes were recorded.

### 2.7. Fecal Analysis

The total dietary (TDF), soluble (SDF), and insoluble (IDF) dietary fiber content of the experimental diet, defatted prior to analysis, were determined following a method described by Asp, Johansson, Hallmer, and Siljeström [20]. The Kjeldahl method was used with a TecatoKjeltec 1026 distilling unit (Tecator AB, Höganäs, Sweden) to determine total fecal nitrogen and protein. Fat content was measured after extracting 5 g with petroleum ether in a Soxtec Avanti 2055 apparatus (FOSS Tecator AB, Höganäs, Sweden). Water content was estimated for all the feces of each rat, collected in glass tubes for ten days. Samples of 2 g were dried in an automatic air exchange dryer at 130 °C until constant weight, expressed as g/100 g of feces. For ash content analysis, 5 g stool samples were weighed into a crucible, which were charred and then burned in a muffle furnace at 550 °C for 6 h until a constant weight was obtained. The average ash content was calculated as g/100 g of feces.

### 2.8. In Vitro Experiments

The in vitro experiments were performed in the Laboratory of Subcellular Structure Analyses of the Institute of Bioorganic Chemistry of the Polish Academy of Sciences in Poznań.

#### 2.8.1. Cell Culture

Normal human lung fibroblasts (MRC-5) were purchased from ECACC, and human skin fibroblasts isolated from a sixty-year-old man with Parkinson’s disease (AG20445) were purchased from the Coriell Institute. The cells were cultured in Eagle’s Minimum Essential Medium (EMEM, Corning) supplemented with antibiotics (Penicillin-Streptomycin Solution (ATCC) and 10% or 15% FBS (Sigma-Aldrich), respectively. Both cell lines were cultured at 37 °C, with the addition of 5% CO_2_ to the atmosphere.

#### 2.8.2. Real-Time Cell Proliferation Analysis by xCELLigence System

Cell proliferation analysis was performed using an xCELLigence system (Roche). The cells were seeded in sixteen-well E-plates (Roche) at a density of 7.5 × 10^3^ per well. After 24 h, the cells were treated with H, NH, and both in final concentrations of 1, 5, 10, 25, 50, and 100 μM. The control cells were cultured in the supplemented medium only. The real-time proliferation of the cells was estimated based on impedance measurement, expressed as a Cell Index (CI) value. The CI was monitored at 30 min intervals from the time of plating for six days.

#### 2.8.3. Confocal Microscopy Analysis of Cell Viability

Cells were seeded on a glass-bottom ten-well CELLview Slide (34 mm^2^, Greiner) at a density of 1 × 10^4^ per well, and incubated until 80% confluency and treated with H, NH, or H/NH in a final concentration of 5, 10, and 25 µM. Cell viability was determined after 24 h using a LIVE/DEAD assay (Thermo Fisher Scientific), following the manufacturer’s protocols. Live cell imaging was performed in FluoroBrite DMEM (Thermo Fisher Scientific) using a Leica TCS SP5 confocal laser scanning microscope with a water/oil immersion objective with 20× magnification and an environmental cell culture chamber. Sequentially scanned images were collected at Ex/Em 495/510–550 nm for living cells and 530/610–650 nm for dead cells. Leica LAS AF and Leica LAS X software with a deconvolution module were used for image processing and fluorescence analysis, respectively.

#### 2.8.4. Apoptosis/Necrosis Assay

The apoptosis/necrosis assay was performed by double staining with CellEvent Caspase 3/7-FITC (Thermo Fisher Scientific) and 7-aminoactinomycin D (BD Pharmigen) fluorescent dyes. Briefly, the cells (4 × 10^5^) were seeded onto six-well plates and incubated until 70–80% confluency. On the next day, the cells were treated with β-carbolines in a final concentration of 5, 10, and 25 µM. Afterward, the cells were detached with trypsin (Thermo Fisher Scientific), washed twice with Dulbecco’s Phosphate Buffered Saline (Thermo Fisher Scientific), and stained with fluorescent dyes for 30 min at 37 °C in the dark, in line with the manufacturer’s protocol. Cells were analyzed immediately with 488 nm excitation using an Accuri C6 flow cytometer (Becton Dickinson).

#### 2.8.5. Cell Cycle Analysis

Next, 5 × 10^5^ cells were seeded on six-well cell culture plates and incubated for 24 h with the compounds for analysis at a concentration of 5, 10, and 25 µM. After incubation, the cells were detached and washed as previously, and the cells were fixed with ice-cold 80% ethanol. After 1 h of incubation at 4 °C, the cells were stored at −20 °C for further analysis. Before analysis, the fixed cells were stained with propidium iodide (PI, 50 μg/mL) with the addition of RNAse A (100 μg/mL) for 30 min at 37 °C in the dark. PI fluorescence was measured using FACSCalibur (Becton Dickinson) and the data were analyzed using FlowJo software.

#### 2.8.6. Mitochondrial Membrane Potential (ΔΨm) Analysis

The cells were seeded at a density of 4 × 10^5^ onto six-well plates for flow cytometry, or at 1 × 10^4^ per well on a glass-bottom ten-well CELLview slide (Greiner) for confocal microscopy, cultured at 37 °C and 5% CO_2_ saturation, and incubated to 70–80% confluency. Subsequently, the cells were treated with 5, 10, and 25 µM of H, NH, or H/NH for 24 h. For cytometric analysis, the cells were detached with trypsin (Sigma-Aldrich) and washed twice with DPBS (Thermo Fisher Scientific). Alterations in ΔΨm were analyzed using the mitochondrial membrane potential sensitive dye 5,5′,6,6′-tetrachloro-1,1′,3,3′-tetraethylbenzimi-dazolylcarbocyanine iodide (JC-1, Thermo Fisher Scientific). Cells were stained with 2.5 µM JC-1 for 30 min at 37 °C in the dark. Immediately after staining, the cells were analyzed using an Accuri C6 flow cytometer (Becton Dickinson) at 488 nm excitation. For the confocal microscopy, the treated cells were stained directly on the CELLview Slides with 5 µM JC-1 for 20 min in growth conditions, then washed twice and placed in FluoroBright DMEM. Live cell imaging was performed with a Leica TCS SP5 confocal laser scanning microscope using a water/oil immersion objective with 20× magnification and an environmental cell culture chamber. Images were collected at Ex/Em 514/529 nm (±20) for J-aggregates (red fluorescence) and 585/590 nm (±20) for JC-1 monomers (green fluorescence). Leica LAS AF and Leica LAS X software with a deconvolution module were used for image processing and fluorescence analysis, respectively. Untreated cells were used as a negative control, with 50 µM CCCP (carbonyl cyanide *m*-chlorophenyl hydrazine) being applied to create a strong, single positive green fluorescence signal.

#### 2.8.7. Total RNA Isolation

Total RNA was isolated using 1 mL of TRI Reagent solution (Thermo Fisher Scientific), following the manufacturer’s protocol. RNA was obtained from the brain tissue of rats fed with chicory extract. Before RNA isolation, the brain tissue was homogenized for 1 min at 30 Hz using a TissueLyser II (Qiagen). DNA residue was removed with DNase I (DNA-free DNA Removal Kit, Thermo Fisher Scientific). The total RNA concentration was measured using a NanoDrop 2000 UV/Vis spectrophotometer at 260 nm.

#### 2.8.8. cDNA Synthesis and Real-Time PCR

Total RNA (0.5 µg) was used for cDNA synthesis with the Transcriptor First-Strand cDNA Synthesis Kit (Roche), following the manufacturer’s description, using oligo(dT) primers. Real-time PCR analysis was performed to assess expression levels of the *BAX*, *BCL2*, *CASP3*, *CASP7*, *LRKK2*, *NDUFV2*, *PARK7 (DJ-1)*, *PGC1*, *PINK1*, *PRKN (PARK2)*, *SIRT1*, *SIRT2*, *SIRT3*, *SIRT6*, *SNCA*, *PRKN*, and *TAU (MAPT)* genes. Each cDNA sample was analyzed using Mono Color Hydrolysis UPL Probes (Roche) selected for each gene using ProbeFinder Software (Roche). The PCR reactions were prepared in line with the manufacturer’s protocol. PCR conditions for all genes were as follows: initial incubation step at 94 °C for 10 min, followed by 40–45 cycles of amplification (15 s at 94 °C, 30 s at 60 °C, and 15 s at 72 °C) (single acquisition), with a final cooling step at 40 °C for 2 min. The analysis was performed using a LightCycler 480 II instrument (Roche). Relative gene expression was calculated using the Roche Applied Science E-Method and normalized to the reference genes *PGK1*, *HPRT1*, and *G6PD*. All standard curves were generated by amplifying a series of two-fold dilutions of cDNA. The primer sequences for the genes being analyzed and UPL (Universal ProbeLibrary, Roche) probes are shown in Table S1.

### 2.9. Statistical Methods

Behavioral tests and fecal analysis data were statistically analyzed using Statistica version 13.1 (StatSoft, Tulsa, OK, USA) and presented as means ± standard deviations (SDs); statistical significance was set at *p* < 0.05. A one-way ANOVA with Tukey’s post hoc test was performed to determine whether there were significant differences. Statistical analysis of in vitro experiment was performed with an unpaired *t*-test using GraphPad Prism 8.00 for Windows. The results are presented as means ± SDs obtained from 6–8 individuals (treated vs. control) and three independent experimental repeats for each one. Two-tailed *p*-values below 0.05 were considered significant.

## 3. Results and Discussion

One of the main features monitored in the animal experiment was the concentration of carbolines in the diet and the blood. The amount of carbolines in the diet was very low, at 0.13 µg/g H and 0.04 µg/g NH. This is much less than in a coffee substitute such as chicory, which has 1.76 µg/g H and 2.90 µg/g NH [21]. Thus, the addition of coffee substitute to the animal diet may increase the concentration of carbolines in the diet.

The in vivo experiment was designed keeping in mind the data from the preliminary in vitro studies with the human spontaneously immortalized keratinocyte cell line (HaCaT), which did not show any toxic effect of β-carbolines in examined range of concentrations, IC_50_ > 100 µM (Figure S1).

We did not observe any effects of the diet on the water, protein, fat, dietary fiber, and ash content of the rats’ feces (Table S2). The average weight gain of rats during the trial (58 g), and the amount of the diet consumed (about 32 g/day) during the experiment, did not differ between the control and experimental groups. This suggests that there was no negative effect on the general health of the animals associated with the addition of carbolines to the diet.

After fourteen weeks of the experiment, the concentration of β-carbolines in the animals’ plasma was measured. These data are presented as concentrations of H and NH in the animals’ blood (Table 1). It can be seen that the level of carbolines in plasma increased with increasing amounts of H or NH in the experimental diet. Taking into account the level of both carbolines in the control diet without carboline additions, H increased by a factor of about four at the greatest addition, while NH increased two-fold at the greatest addition; however, the final concentration of NH was greater than H, as is typical of natural products [13].

**Figure 1:**
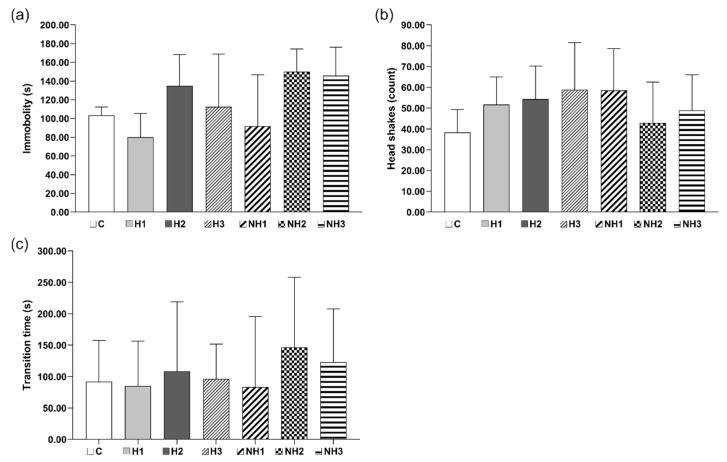
Behavioral tests: (**a**) Porsolt test: mean time of immobility, (**b**) Porsolt test: mean number of head shaking, (**c**) orientation and spatial memory test: mean time transition through the maze. Samples: C = control, H1 = 10 µg H/kg, H2 = 15 µg H/kg, H3 = 20 µg H/kg, NH1 = 8 µg NH/kg, NH2 = 12 µg NH/kg, NH3 = 16 µg NH/kg. Number of animals in all groups *n* = 6.

Despite the above, the battery of behavioral tests in the first stage of the experiment did not reveal any statistically significant differences in animal activity; however, we did note some positive animal reactions in the Porsolt test (Figure 1), where animals supplemented with carbolines displayed more frequent head movements than those in the control group. Moreover, the rats that received the lowest amounts of H and NH were more mobile than the control animals, than the rats fed greater amounts of β-carbolines. The behavioral effects induced by β-carbolines observed in our rats are in agreement with the literature data, which supports the antidepressant action of those compounds. For example, in the study of Farzin and Mansouri [8], treatment with carbolines reduced the immobility time in the forced swimming test.

Studies on human cell lines were also undertaken to show the effects of β-carbolines on cell viability and intracellular processes. The effect of H and NH was examined using aged normal human cells (MRC-5 fibroblasts, ATCC, passage 17–20) and cells isolated from a patient with Parkinson’s disease (AG20445 fibroblasts, Coriell). Since immortalized cells often exhibit cancer cell characteristics, the HaCaT cell line used in the preliminary analysis was excluded from further studies.

To determine the viability of the treated cells, we performed real-time cell proliferation analysis using the xCELLigence system, which allows continuous intravital analysis of adherent cell cultures [22]. Changes in the impedance directly proportional to the proliferation rate (viability) were monitored during cell culturing in the presence of H, NH, or H/NH at final concentrations of 1, 5, 10, 25, 50, and 100 µM.

**Figure 2:**
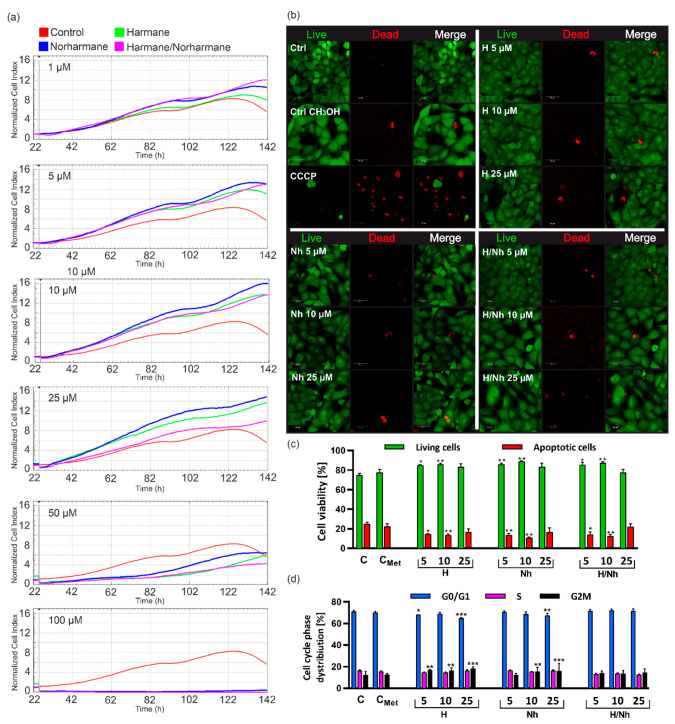
Viability analysis of aged human cells after harman and norharman treatment. (**a**) Real-time analysis of MRC-5 cells (passage 19, aged cells) proliferation by the xCELLigence system for 6 days in the presence of 1, 5, 10, 25, 50, and 100 µM harmane (H), norharmane (NH) or a mix of both (H/NH). Proliferation was monitored in 30 min intervals. Vertical lines on the graphs indicate the time points for adding compounds. Normalized Cell Index (NCI) = CI_original_/CI_normalize time_. (**b**) Confocal microscopy analysis of MRC-5 viability using LIVE/DEAD assay kit. Panels with green (Ex/Em 495/515 nm) and red fluorescence (Ex/Em 530/635 m) show live and dead cells, respectively. Merged images are presented on the right panel. (**c**) Flow cytometry analysis of apoptosis in MRC-5 cells treated with 5, 10, and 25 µM after 24 h with Casp 3/7-FITC/7-AAD dual staining. (**d**) Flow cytometry analysis of H, NH, and H/NH effect (5, 10, and 25 µM) of on the cell cycle phase distribution in MRC-5 cells after 24 h. The data are presented as the mean ± SEM (standard error of the mean) from three independent experiments. Statistical significance (ANOVA): (*) *p* < 0.05, (**) *p* < 0.01, (***) *p* < 0.001.

We found out that, in the low concentration range (1–25 µM), neither H nor NH showed any cytotoxic effect on the MRC-5 cell line. Moreover, 24 h after the administration of both compounds, we noted a significant increase in the cell proliferation rate, as compared to the control, and this effect was observed throughout the experiment (Figure 2a).

The greatest proliferation rate was obtained for the NH and H/NH mix used at final concentrations of 1, 5, and 10 μM. For 25 μM of H/NH mix, a significant decrease in the proliferation level was observed compared to the results received for H or NH, used separately, and remained on the control cell level. A toxic effect of the β-carbolines was noted at a concentration of 50 µM, where the proliferation of MRC-5 was reduced by approximately 50%. However, concentrations of 100 µM and higher completely inhibited cell growth under experimental conditions. A similar effect was obtained for AG20445 (PD cells) (Figure S2a). A significant increase in CI values was noted 24 h after treatment with the range of 1–50 µM of H, NH, and H/NH. Over the next 24 h, the proliferation rate remained at the same level in these concentrations. However, an opposite effect was observed for 50 µM of the H/NH mix, where the proliferation rate decreased below the level of control cells. We found that total inhibition of AG20445 proliferation occurred at concentrations above 100 µM, as was observed for MRC-5 cells. In other studies, the cytotoxicity of natural carbolines and their modified synthetic analogs was analyzed in other cell lines, especially of tumor origin, to investigate their anticancer activity [23,24,25]. Previous results reported for harman, expressed as ED_50_ values (effective dose for 50% of the population), were determined for tumor cell lines, including KB, A549, CAKI-1, MCF-7, IA9, SK-MEL-2, and U-87-MG as well as HEL and were 8.9, 9.3, 8.0, 19.0, 6.1, >20, 19.0, and 9.8 µg/mL, respectively [25]. The 50% decrease in MRC-5 cell viability at a concentration of ~50 µM, which corresponds to 9.11 μg/mL (harman) and 8.41 μg/mL (norharman), is a result comparable to previous ones. It was shown in anticancer studies that modification of β-carboline alkaloids could impact an increase of their cytotoxicity [23,24,25].

In the next step, we performed flow cytometric analysis of cell apoptosis after 24 h of incubation in the presence of H, NH, and H/NH (5, 10, and 25 µM) (Figure 2c and Figure S2b). We showed that these compounds do not induce apoptosis under the experimental conditions. Furthermore, the β-carbolines led to an increase in the percentage of live cells, as compared to the untreated cells, especially at the final concentrations of 5 and 10 µM (Figure 2c and Figure S2b). These results were confirmed by confocal microscopy analysis using a live/dead fluorescent test (Figure 2b). In the previous research, Hamman et al. [26] demonstrated that synthetic methylated derivative of norharman (i.e., 9-methyl-β-carboline (9-me-BC)), discovered as a neuroprotective compound, reduced the number of necrotic cells in the primary mesencephalic dopaminergic cell culture derived from embryonic mice as a model with relevance to Parkinson’s disease, which corresponds to our results. Moreover, 9-me-BC was identified as an agent capable of increasing the number of dopaminergic neurons in a concentration-dependent manner while maintaining the functionality of these neurons [26,27]. On the other hand, dimethyl β-carboline derivatives (2,9-dimethyl-BC) exert highly toxic effects not only on dopaminergic neurons but also on other cell constituents in primary dopaminergic culture [28].

Cytometric analysis of DNA content in cells treated with 5, 10, and 25 µM of H, NH, or H/NH allowed us to assess their effect on the cell cycle. We found that these compounds regulate the cell cycle of both the aged MRC-5 and AG20445 cells. We noted an increase in the percentage of cells in G2M phase after β-carboline treatment (Figure 2d and Figure S2c). The G2 phase prepares the cells for the mitosis process, which occurs in the M phase [29]. The results of the cell cycle analysis correlate with data from the cell viability analysis and may explain the increased percentage of live cells in the apoptosis test.

**Figure 3:**
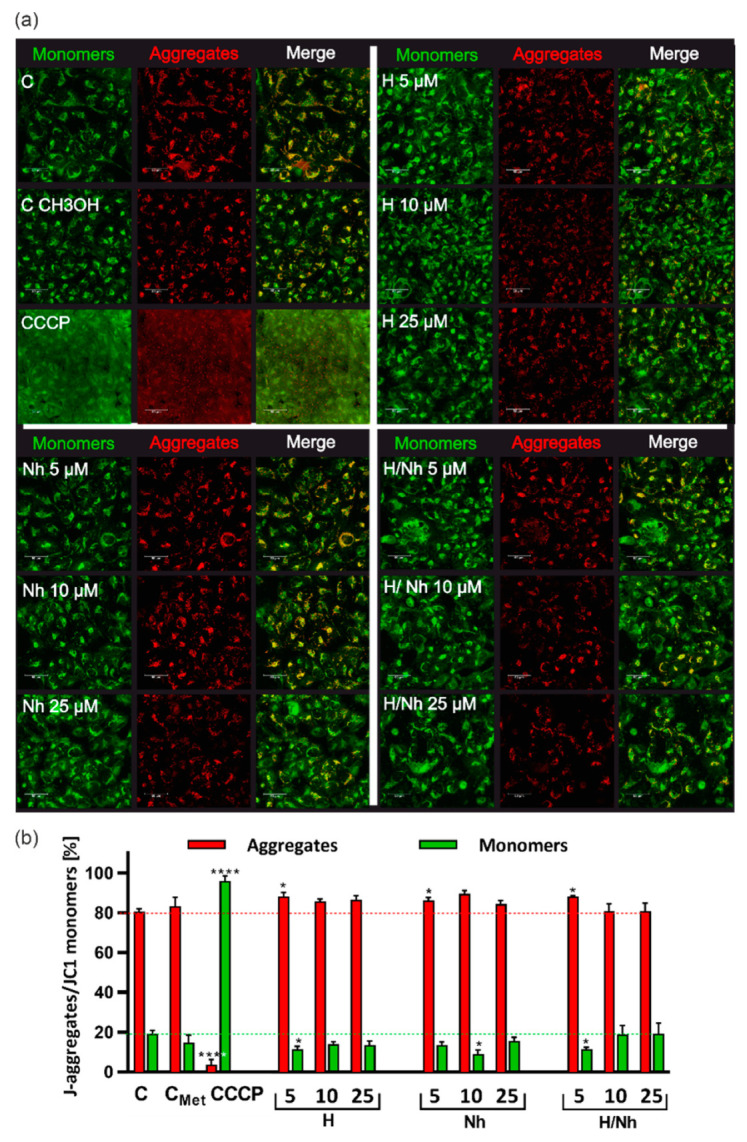
Mitochondrial oxidative stress analysis after harmane and norharmane treatment. (**a**) Confocal microscopy analysis of the mitochondrial membrane status in MRC-5 after 24 h of harmane (H), norharmane (NH), and mix of both (H/NH) treatment (5, 10, and 25 µM) determined by JC-1 staining. Left panels show JC-1 monomers (green fluorescence, Ex/Em 485/530 nm) which indicate a decrease of mitochondrial potential and the middle panels present the J-aggregates (red fluorescence, Ex/Em 535/590 nm) which indicate high membrane potential. Merged images are on the right panels. (**b**) Flow cytometry analysis of changes in the mitochondrial membrane potential (Ψm). The fluorescence intensity histograms are representative of three independent experiments, whereas the bar graphs represent the mean fluorescence intensity ± SEM. Statistical significance (ANOVA): (*) *p* < 0.05, (**) *p* < 0.01, (***) *p* < 0.001, (****) *p* < 0.0001. CCCP (50 μM) was used as a positive control to decrease Ψm.

The effects of H and NH on mitochondrial redox state were also investigated. One factor associated with aging and the progression of certain cell neurodegenerative changes is a chronic state of oxidative stress [30]. The mitochondria play a crucial role in cell viability. Alterations in their activity affect the function, metabolism, and the lifespan of cells [31]. Fundamental cellular functions, such as ATP generation, Ca^2+^ uptake and storage, and the generation and detoxification of reactive oxygen species (ROS), are driven by the mitochondrial membrane potential (ΔΨm). This parameter is a reliable measure of cell stress and apoptosis [32]. We evaluated the mitochondrial redox state of MRC-5 and AG20445 cells after 24 h incubation with 5, 10, and 25 µM of H, NH, and H/NH using confocal microscopy and flow cytometry (Figure 3 and Figure S3).

For this purpose, cells were stained with JC-1 cationic carbocyanine dye. We demonstrated that both β-carbolines improved the energetic state of the cells under experimental conditions, as seen by the increasing percentage of cells with high ΔΨm in the study population, as compared with the control (Figure 3b and Figure S3b). This result indicates the good physiological condition of the treated cells and shows that β-carbolines do not affect mitochondrial ROS production. Furthermore, the improved energetic state of the treated cells may suggest the antioxidant effects of analyzed β-carbolines used in low concentrations (Figure 3b and Figure S3b). The antioxidative action of β-carbolines, whether natural or synthetic, against reactive forms of oxygen has been demonstrated in the study of Lehmann [33]; there is no other evidence in the literature concerning this aspect of neurodegenerative protection. However, it was shown that harman and its derivatives have a significant protective effect against H_2_O_2_ and paraquat oxidative agents in yeast and mammalian cells, and that their ability to scavenge hydroxyl radicals contributes to their antimutagenic and antigenotoxic effects [34]. Moreover, β-carboline alkaloids activated expression of the antioxidant enzymes superoxide dismutase (SOD) and glutathione peroxidase (GSH-px) and suppressed the formation of maleic dialdehyde (MDA) in the cortex of mice with induced dementia and improved the ability of antioxidant defense, indicating their neuroprotective effects [35]. Nevertheless, other studies have shown that a dimethylated β-carboline derivative such as 2,9-dimethyl-BC increased free radical production, decreased respiratory activity and mitochondrial membrane potential as well as ATP content, contributing to the apoptotic mode of cell death [28].

Two groups of animals were used in stage 2 of the in vivo experiment: one group received the control diet, and the second received the experimental diet enriched with coffee substitute (chicory), and with the β-carboline quantities matched to the quantities found in two cups of chicory coffee, as might be consumed daily by humans. We chose a two-cup dose on account of the positive observation made in the first stage of the experiment for the lowest doses of carbolines, as well as suggestion from in vitro studies done in parallel, as described above. The main concept of stage 2 of the experiment was to use a natural source of β-carbolines in the diet and to observe its effect on the rats’ behavior. The richest natural source of β-carbolines is coffee and its substitutes. In our previous studies [16], we noted that roasted artichoke contained very significant levels of carbolines, and we proposed a mixture of chicory and artichoke to give the highest possible concentration of β-carbolines in coffee substitute. However, we ultimately omitted the artichoke as we found it contains relatively high levels of some toxic compounds, particularly of acrylamide [21]. During the experiment, the body weight of the rats and the amount of diet they consumed were monitored; they proved to be similar in both groups. Various nutritional parameters determined from the feces also did not differ (Table S2). After fourteen weeks of the experiment, the concentration of carbolines in the animals’ blood was measured using the method described in Section 2 and presented in Table 1. The literature does not have much data on the level of carbolines in blood. Our data confirmed the natural presence of β-carbolines in the control samples. The level of NH in the experimental animals’ blood samples was about three times higher than that of H (by comparison, NH and H occurred at 0.060 and 0.020 ng/mL, respectively in the control). The concentration of both carbolines was higher in the plasma of rats receiving a diet enriched with coffee substitutes than in the control group.

Li et al. [9] found significantly higher levels of β-carbolines in rat blood than in our experiment; however, the procedures in the experiments differed greatly and the results are thus almost impossible to compare. In our experiment, the samples of blood were taken after feeding period, in which the experimental diet provided a constant supply of carbolines, while in the study of Li et al. [9] the carbolines were administered either orally or by intravenous injection, and plasma was taken intravitally. Li et al. [9] showed how the large quantity of carbolines injected into the animal decreased rapidly in the plasma over just a few hours; however, they were using very large doses of the size employed in drug therapy: the level of H in the plasma was initially about 1000 mg/mL, and immediately decreased. In our experiment, carboline levels in plasma must obviously have decreased, but we made use of small doses that could be taken in as part of the everyday diet. The measurements of carboline levels in the blood, and their proportional growth in parallel with their growth in the diet is only evidence of the metabolizing of these compounds after eating. Taking into account the average body mass of animals, their average feed consumption, and the concentration of carbolines in the feed, only about 0.15% of the H and about 0.5% of NH taken in was found in the blood. This led to a small increase in the level of carbolines in the rats’ blood, which nonetheless apparently sufficed to affect the rats’ behavior. As compared with the level of carbolines in human blood, which was 4.1 ± 9 pg/mL for H and 4.9 ± 7.9 pg/mL for NH [36], or in other papers for norharman 26.9 ± 10.7 pg/mL [37], our values were different, though of a similar magnitude.

**Figure 4:**
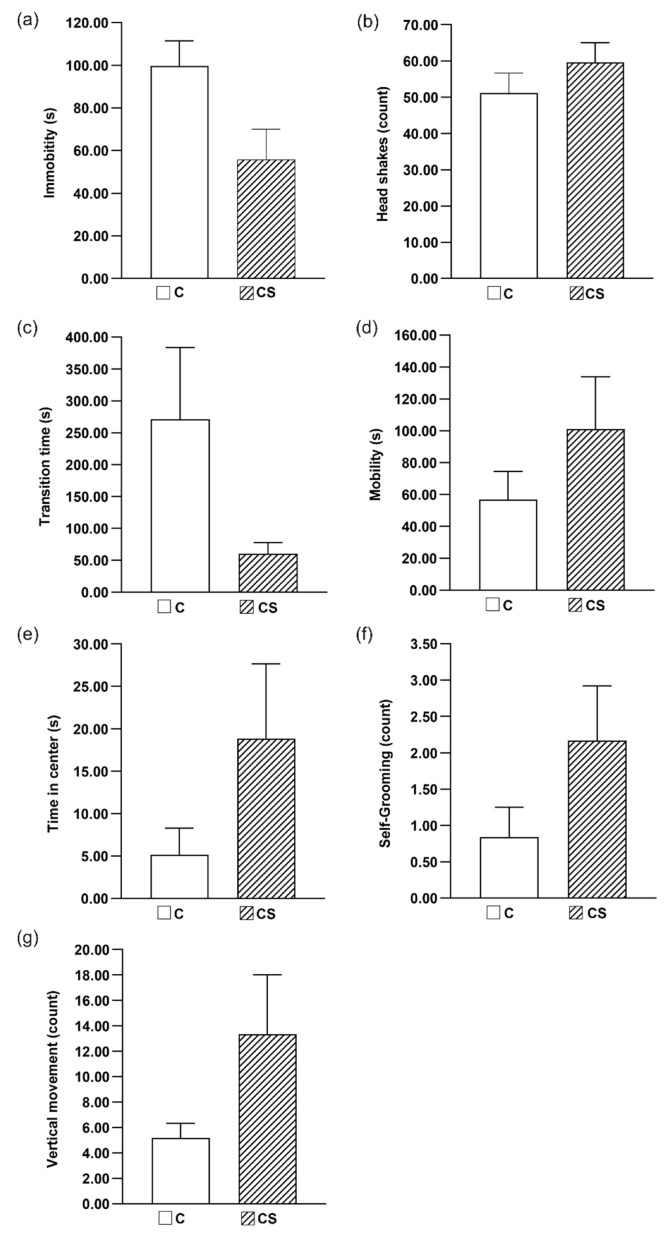
The battery of behavioral tests: (**a**) Porsolt test: mean time of immobility, (**b**) Porsolt test: mean number of head shaking, (**c**) classical labyrinth test: mean time transition through the labyrinth, (**d**) open field test (OFT): mean time of mobility, (**e**) OFT: mean time in center, (**f**) OFT: mean number of self-grooming (facial wash, body cleaning, genital grooming), (**g**) OFT: mean number of vertical movement. C = control group, CS = group with 12.5 g of chicory/kg of exp. diet. Number of animals in all groups *n* = 6.

The activity of the animals, as measured by the series of behavioral tests, is presented in Figure 4. It can be seen from the data that the addition of the coffee substitute to the animal diet made the rats more active; in this case, all the observations from behavioral tests were statistically significant. This may suggest a possible positive effect of carbolines in the diet on animal behavior pattern. In all the behavioral experiments, it was seen that ingestion of a diet enriched with coffee substitute reduced immobility time during FST and transition time via labyrinth, while increasing mobility time, time spent in the center, and frequencies of vertical movements and self-grooming of rats during OFT. It should thus be highlighted that this is the first paper to describe a positive effect of carbolines from coffee substitute on the behavioral pattern of rodents. It is also important to mention, as in stage 1, the tested diet had no adverse effects on body weight or fecal parameters. In previous research Gruss et al. [38] demonstrated that synthetic methylated derivative of harmane (9-me-BC) acts as a cognitive enhancer in a hippocampus-dependent task. Treatment of rats with 9-me-BC showed significantly accelerated acquisition compared with controls. It was concluded that the behavioral effects may be associated with a stimulatory impact on hippocampal dopamine levels and dendritic and synaptic proliferation.

The data from both stages show that increasing amounts of β-carbolines in the animal diet were reflected in their increasing concentrations in the blood, as measured after fourteen weeks of the experiments. It is worth underlining that the amount of carbolines added to the diet was very low in comparison to that reported by other authors [9]. However, stage 1 of the experiment gave no real evidence of any effect of these additions on animal activity. Further studies are needed to explain this; currently, we can only suggest that this is due to the natural origin of the β-carbolines used in stage 2, or the occurrence of both carbolines in the diet. In stage 1 of the animal experiment, they were used as individual, standard chemical components.

**Figure 5:**
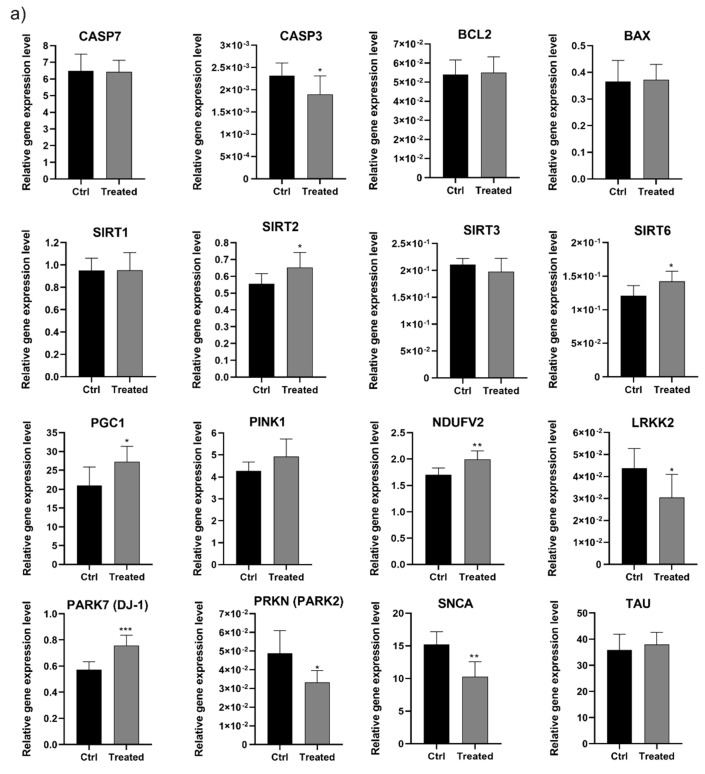

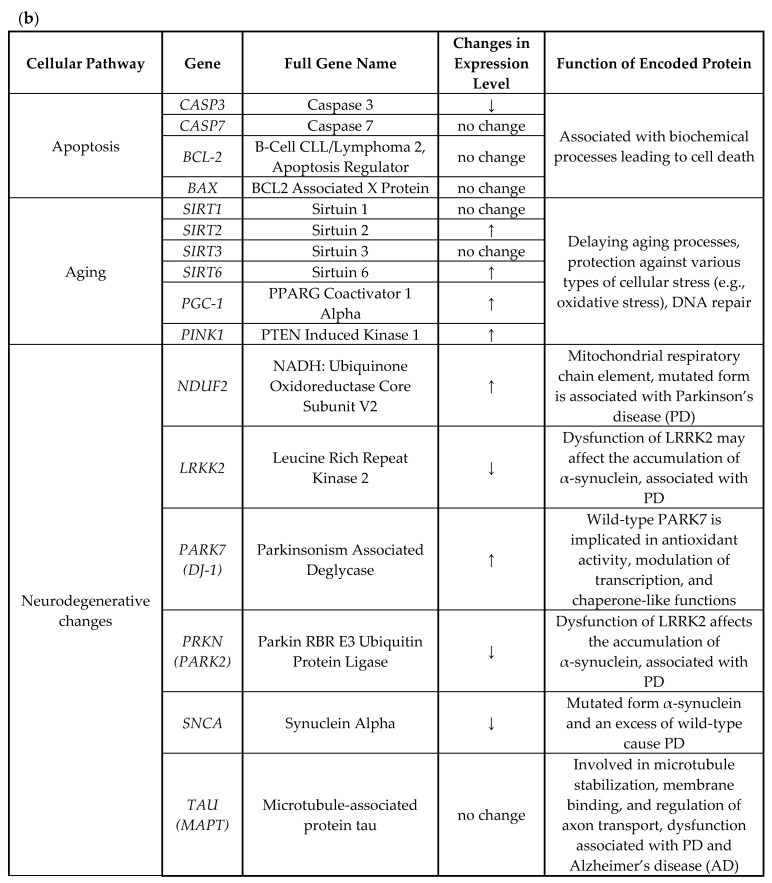
The expression level of selected genes in the brain tissue of aged rats fed with chicory extract-enriched feed. (**a**) Relative real-time PCR analysis of pro-apoptotic genes (CASP3, CASP7, BAX, BCL2), genes involved in delaying aging processes, different cellular type of stress, as well as DNA repair processes (SIRT1, SIRT2, SIRT3, SIRT6, PINK1, PGC1), and genes associated with neurodegenerative diseases (SYNC, LRKK2, PARK7, PRKN, TAU). The results are presented as the mean ± SD obtained from 6–8 individuals (treated vs. control) and three independent experimental repeats for each one. Statistical significance (*t*-test). Two-tailed *p*-values below 0.05 were considered statistically significant. (*) *p* < 0.05, (**) *p* < 0.01, (***) *p* < 0.001 (**b**) Table summarizing the analyzed genes and the function of encoded proteins, as well as their involvement in main cellular processes. Up arrow (up-expression); Down arrow (down-expression).

We also examined the effect of β-carbolines on the expression level of selected genes associated with aging, apoptosis, and neurodegenerative diseases (including Parkinson’s) in the brain tissue derived from rats fed chicory extract as a source of natural β-carbolines. Expression levels were determined using real-time PCR (Figure 5). The genes we examined were of three types: apoptotic genes (caspase 3, 7, BAX, BCL2), whose expressions are associated with biochemical processes leading to cell death [39]; genes involved in delaying aging processes and protecting the cell from various types of stress, such as oxidative stress, as well as those involved in DNA repair processes (SIRT1, 2, 3 and 6, PGC-1α, PINK1) [30,40,41]; and genes associated with neurodegenerative diseases, such as LRKK2, SYNC, PARK7 (DJ-1), PRKN (PARK2), and TAU [42,43].

We discovered that the diet rich in β-carbolines caused a significant decrease in caspase-3 (CASP3) expression level of about 20%, while the expression of the other analyzed genes involved in apoptosis remained on the level of the control animals. CASP3 is an effector factor responsible for the proteolytic cascade leading to cell death [39]. A similar effect in a decrease of caspase-3 activity was observed for the primary mesencephalic dopaminergic cell culture derived from embryonic mice after treatment with 9-methyl-β-carboline (a methylated derivative of norharmane) [26]. It can be assumed that the obtained result is a protective effect of β-carbolines. Moreover, studies on 9-me-BC revealed that β-carboline downregulated the expression of other proteins in apoptosis-relevant pathways like FAS, Gadd45a, and Hspb1, as well as reduced the expression of inflammation-related genes like Cxcl9, Irf1, Fasl, Icam1, Tnf, and Vcam1 which are also relevant for apoptosis [26]. In the second group of genes, we noted an increase in the expression of SIRT2 (~17%) and SIRT6 (~12%), while the expression of SIRT1 and SIRT3 remained at the level of the control (Figure 5). It is known that sirtuins are involved in delaying the aging process [40]. SIRT2 upregulates the expression of FOXO3 target genes, thus decreasing ROS level [44], whereas SIRT6 plays essential roles in metabolic homeostasis, stress responses, genomic stability, and DNA repair [45]. Furthermore, SIRT6, a key modulator of the NFκB pathway, is involved in slowing the aging process [45]. According to Kanfi et al. [46], overexpression of SIRT6 in male mice significantly extends their life.

A significant increase in PGC-1α (~30%) and PINK1 (~17%) expression levels was also observed in the brains of treated rats. It is widely accepted that elevations in the level of PGC-1α—a critical regulator of mitochondrial energy metabolism and biogenesis that is downregulated in PD brains—leads to protection against α-synuclein-induced neurodegeneration in cell models [42]. PGC-1α induces the expression of many ROS-detoxifying enzymes that protect neural cells from oxidative-stressor-mediated cell death [41], whereas PINK1 is a neuroprotector involved in preventing mitochondrial damage and promotes cell survival [30]. Moreover, PINK deficiency is associated with mitochondrial dysfunction, as well as with increased oxidative cellular stress and subsequent neuronal cell death [30].

We also observed significant differences in the expression of the third group of genes—those associated with neurogenerative changes. Decreases in LRRK2, SNCA, and PRKN (PARK2) of about 30%, 32%, and 18% were observed, while PARK7 (DJ1), NDUFV2, and TAU expression increased by 30%, 20%, and 5%, respectively (Figure 5). It is known that the dysfunction of proteins encoding these genes is associated with the development and progression of neurodegenerative diseases, including Parkinson’s disease [47]. The LRRK2, SNCA, and PRKN genes are the key factors influencing PD. It has been reported that dysfunction of LRRK2 may affect the accumulation of α-synuclein and its pathology, altering cellular functions and signaling pathways though the kinase activation of LRRK2 [48]. On the other hand, the loss of parkin (PRKN) activity is the second most commonly known cause of PD [49]. However, in our research on experimental rats without a diagnosis of neurodegenerative disease, the expression level of the selected genes is related to the natural aging process and diet. The results should, therefore, be interpreted bearing in mind the physiological significance of the encoded proteins. Wild-type parkin plays an important role in mitochondrial quality control and turnover and promotes autophagy and the selective elimination of impaired mitochondria [49]. The precise physiological function of LRRK2 remains largely unknown, though recent studies have indicated that it is involved in cellular functions such as cytoskeletal maintenance, vesicle trafficking, autophagic protein degradation, and the immune system [48]. Some data have shown that not only the expression of the mutated form α-synuclein, but also an excess of wild-type, can cause Parkinson’s disease [42]. This contributes to abnormal protein aggregation, mitochondrial abnormalities, increased levels of ROS, and enhanced susceptibility to apoptosis [47]. Thus, the observed decrease in SNCA expression level may be seen as a beneficial result of enriching the diet with β-carboline-rich chicory extract. The upregulation of the PARK7 expression may also be seen as a positive result. It has been shown that wild-type PARK7 is implicated in antioxidant activity, modulation of transcription, and chaperone-like functions [50]. Moreover, PARK7 increases VMAT2 expression and function, protecting cells against dopamine toxicity and oxidative stress [51]. The other examined NDUFV2 gene, which was also overexpressed, is involved in controlling the stabilization of the assembly and optimal electron transport inside complex I, and also might act as an antioxidant electron carrier. Defects in NDUFV2 are associated with neurodegenerative disorders, including PD, but there is no literature data on NDUFV2 impairment during normal brain aging [52]. The minimal change in the expression level was observed for the gene encoding the TAU protein, which is involved in microtubule stabilization, membrane binding, and regulation of axon transport [43].

The data from gene expression studies correlate with the results of behavioral experiments, which show that treated rats were in significantly better condition than the control animals. These results may be considered an effect of the positive impact of a diet enriched with β-carbolines on aged individuals. Some literature data reported that 9-me-BC lowered the content of α-synuclein in the primary dopaminergic cell cultures [27], as well as stimulated the gene expression of several important neurotrophic factors like Artn, Bdnf, Egln1, Tgfb2, and Ncam1, which are known to stimulate neurite outgrowth and exhibit neuroprotective and neuroregenerative properties to dopaminergic neurons [53].

Taken together, the presented results and literature data on neuroprotective and neuroactive effects suggest that further research is required to better understand the importance of β-carbolines in the aging process and neurodegeneration.

## 4. Conclusions

The addition of individual chemical standards of the β-carbolines harman and norharman to the diet of rats did not produce a very marked effect on the animals’ behavior; however, they were transmitted to the blood in proportion to the amount eaten. The animal diet enriched with coffee substitute (chicory), a natural source of carbolines, resulted in a higher concentration of harman and norharman in the blood and had a positive effect on the animals’ activity. The benefits of using a diet rich in β-carbolines were clearly visible in the results of behavioral studies on the rats, the increase in the level of harman and norharman in the blood plasma, and the results of the in vitro studies—particularly in terms of the gene expression levels in brain tissue of treated rats. We observed upregulation of genes associated with delaying aging processes and protecting oxidative stress but did not notice changes in expression of genes associated with processes leading to apoptosis. Furthermore, we reported a decrease of expression in the level of PD risk genes (*PARK2*, *SNCA*). The results obtained in the in vitro study on cell lines showed a protective effect of harman and norharman on aged human cells. These encouraging results provide the basis for further research into this subject.

## Figures and Tables

**Table 1:** Contents of harman (H) and norharman (NH) in animal blood: stage I and II (ng/mL).

Animal Group	Harman (ng/mL)	SD	Norharman (ng/mL)	SD
	Stage I			
C	0.020	0.003	0.060	0.004
H1	0.038	0.004	0.049	0.006
H2	0.052	0.010	0.054	0.011
H3	0.084	0.009	0.046	0.005
NH1	0.018	0.008	0.080	0.039
NH2	0.021	0.010	0.089	0.013
NH3	0.018	0.005	0.107	0.022
	Stage II			
C	0.010	0.001	0.067	0.007
CS	0.016	0.004	0.110	0.014

Stage I: LOD (limit of detection): H (0.013 ng/mL), NH (0.067 ng/mL); LOQ (limit of quantification): H (0.026 ng/mL), NH (0.137 ng/mL); C = control; H1 = 10 µg H/kg of exp. diet, H2 = 15 µg H/kg of exp. diet; H3 = 20 µg H/kg of exp. diet; NH1 = 8 µg NH/kg of exp. diet, NH2 = 12 µg NH/kg; NH3 = 16 µg NH/kg. Stage II: LOD: H (0.009 ng/mL), NH (0.044 ng/mL); LOQ: H (0.018 ng/mL), NH (0.088 ng/mL); C = control; CS = 12.5 g of chicory/kg of exp. diet. Number of animals in all groups *n* = 6.

## Supplementary Materials

Supplementary materials can be found at https://www.mdpi.com/1422-0067/21/15/5245/s1. Figure S1. MTT assay of harman and norharman cytotoxicity on HaCaT cells at various concentrations; Figure S2. Analysis of PD human cells viability after harmane and norharmane treatment. (**a**) Real-time analysis of AG20445 cells proliferation by the xCELLigence system for 6 days in the presence of 1, 5, 10, 25, 50, and 100 µM harmane (H), norharmane (NH) or a mix of both (H/NH). Proliferation was monitored in 30 min intervals. Vertical lines on the graphs indicate the time points for adding compounds. (**b**) Flow cytometry analysis of apoptosis in AG20445 cells treated with 5, 10, and 25 µM after 24 h with Casp 3/7-FITC/7-AAD dual staining. (**c**) Flow cytometry analysis of cell cycle in AG20445 after 24 h of H, NH, and H/NH treatment (5, 10, and 25 µM) The data are presented as the mean ± SEM from three independent experiments. Statistical significance (ANOVA): (*) *p* < 0.05, (**) *p* < 0.01, (***) *p* < 0.001; Figure S3. Mitochondrial oxidative stress analysis after harmane and norharmane treatment. (**a**) Confocal microscopy analysis of the mitochondrial membrane status in AG20445 after 24 h of harmane (H), norharmane (NH), or a mix of both (H/NH) treatment (5, 10, and 25 µM) determined by JC-1 staining. Left panels show JC-1 monomers (green fluorescence, Ex/Em 485/530 nm) which indicate a decrease of mitochondrial potential and middle panels present the J-aggregates (red fluorescence, Ex/Em 535/590 nm) which indicate high membrane potential. Merged images are on the right panels. (**b**) Flow cytometry analysis of changes in the mitochondrial membrane potential (Ψm). The fluorescence intensity histograms are representative of three independent experiments, whereas the bar graphs represent the mean fluorescence intensity ± SEM. Statistical significance (ANOVA): (*) *p* < 0.05, (**) *p* < 0.01, (***) *p* < 0.001. CCCP (50 μM) was used as a positive control to decrease Ψm; Table S1. List of primers and UPL probes used for real-time PCR analysis; Table S2. Mean content of fecal factors in 100g: insoluble dietary fiber (IDF), soluble dietary fiber (SDF), protein, fat, ash and water. Stage I and stage II.
