# Supplementary figures and images for: β-Carbolines in Experiments on Laboratory Animals
Source: Int J Mol Sci. 2020 Jul 24;21(15):5245. doi: 10.3390/ijms21155245 (PMC7432475; doi:10.3390/ijms21155245)

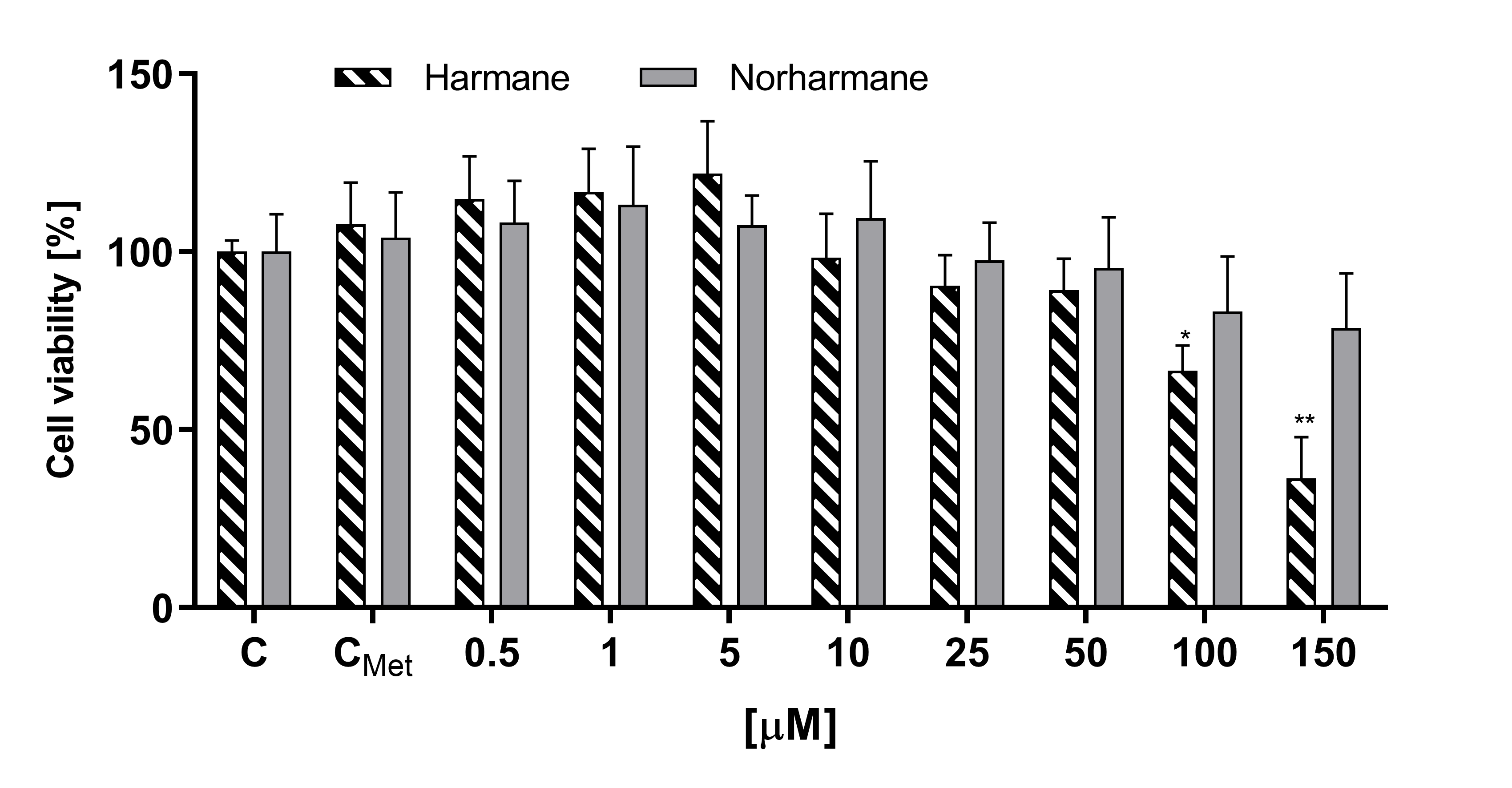

Supplement: Supplementary file 1 [file ijms-21-05245-s001.zip › ijms-859032-supplementay/Supplementary Figure S1.tif]

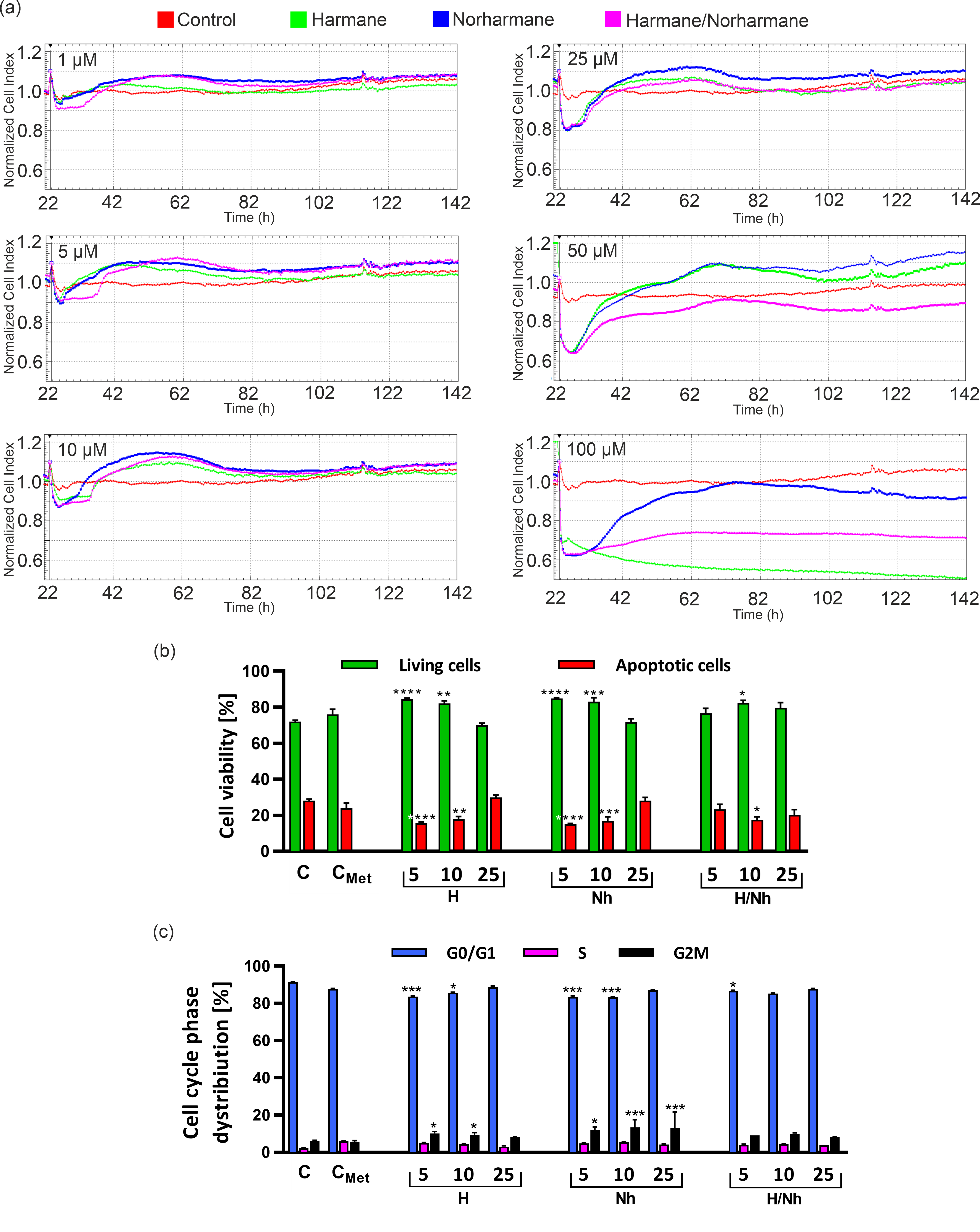

Supplement: Supplementary file 1 [file ijms-21-05245-s001.zip › ijms-859032-supplementay/Supplementary Figure S2.tif]

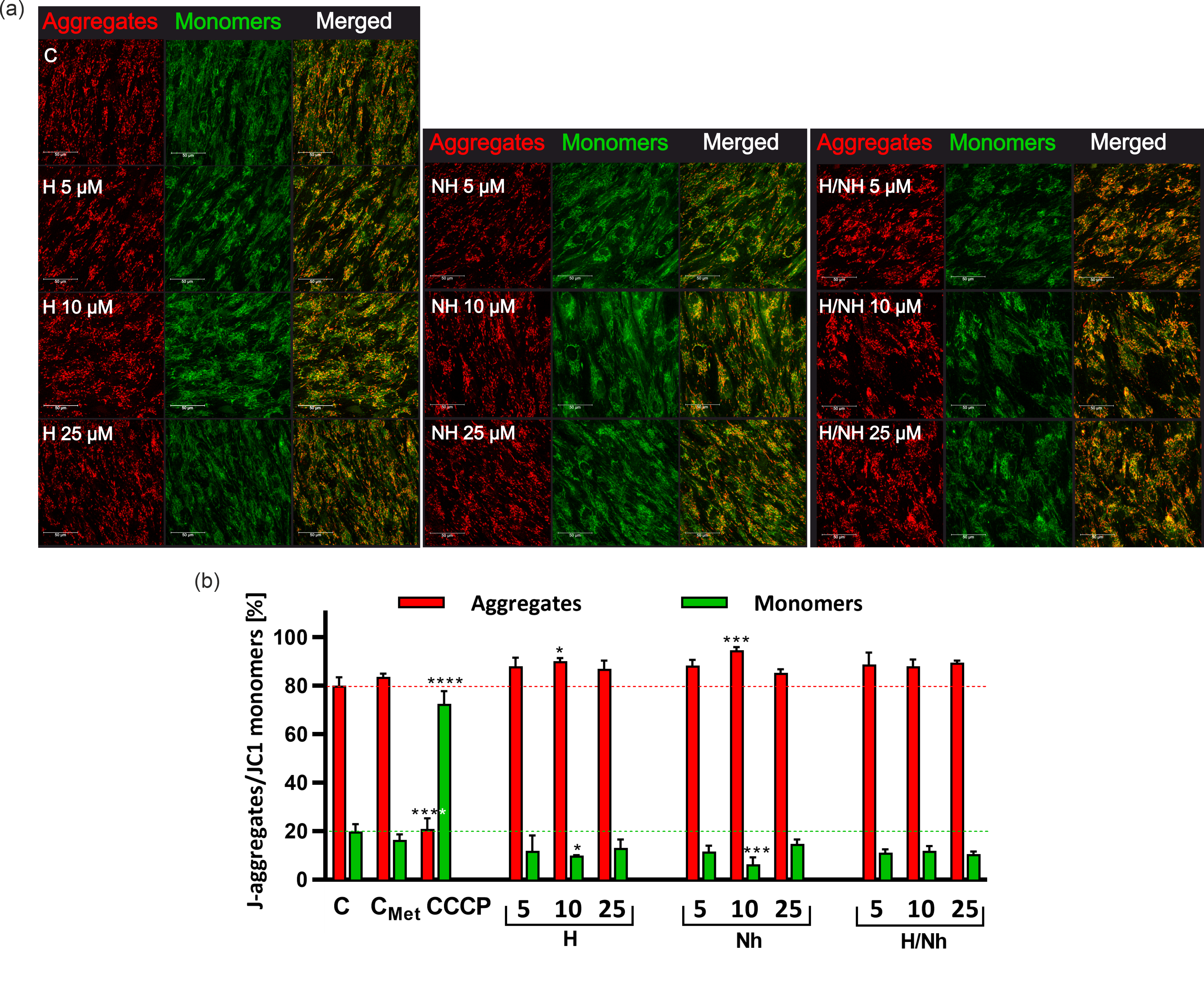

Supplement: Supplementary file 1 [file ijms-21-05245-s001.zip › ijms-859032-supplementay/Supplementary Figure S3.tif]
