# Supplementary material for: β-Carbolines in Experiments on Laboratory Animals
Source: Int J Mol Sci. 2020 Jul 24;21(15):5245. doi: 10.3390/ijms21155245 (PMC7432475; doi:10.3390/ijms21155245)
Supplement: Supplementary file 1 [file ijms-21-05245-s001.zip › ijms-859032-supplementay/ijms-859032-supplementary Table S1 after proof.doc]

Table S1. List of primers and UPL probes used for real-time PCR analysis.

| **Gene shortcut** | | **Full gene name** | **Forward primer (5’-3’)** | **Reverse primer (5’-3’)** | **UPL no.** |
| --- | --- | --- | --- | --- | --- |
| Reference  genes | *PGK1* | Phosphoglycerate Kinase 1 | aaccagataacgaataaccaaagg | gctccattgtccaagcaga | 66 |
|  | *HPRT* | Hypoxanthine Phosphoribosyltransferase 1 | ggtccattcctatgactgtagatttt | aacaatcaagacgttctttccag | 22 |
|  | *G6PD* | Glucose-6-Phosphate Dehydrogenase | ttatcatcatgggtgcatcg | aaggtgtcttcgggtagaagg | 83 |
| Target  genes | *BAX* | BCL2 Associated X Protein | cgagctgatcagaaccatca | ggggtcccgaagtaggaa | 69 |
|  | *BCL2* | B-Cell CLL/Lymphoma 2, Apoptosis Regulator | gtacctgaaccggcatctg | ggggccatatagttccacaa | 75 |
|  | *CASP3* | Caspase 3 | aaacctccgtggattcaaaa | agcccatttcagggtaatcc | 56 |
|  | *CASP7* | Caspase 7 | tgcaggatctgcttagacga | gccgtctttcccataaatca | 18 |
|  | *LRRK2* | Leucine Rich Repeat Kinase 2 | caagagggacgaggagattg | gtgacggccagagaatgc | 84 |
|  | *NDUFV2* | NADH:Ubiquinone Oxidoreductase Core Subunit V2 | tgggtggctgcctatctct | gactctcattggaggcacttg | 29 |
|  | *PARK7 (DJ-1)* | Parkinsonism Associated Deglycase | ggaccatacgatgtggttgtt | ccttcaccaaagccgactc | 79 |
|  | *PRKN (PARK2)* | Parkin RBR E3 Ubiquitin Protein Ligase | tggacacagacagcaagagtg | tgtggtaggtgggtttagctg | 71 |
|  | *PGC1α* | PPARG Coactivator 1 Alpha | ttgacagtggagctttgtgg | gggcttatatggaggtgtgg | 18 |
|  | *PINK1* | PTEN Induced Kinase 1 | tcgtctcaaagggagcagat | ttaagatggcttcgctggag | 1 |
|  | *SIRT1* | Sirtuin 1 | aagaccagtagcactaattccaagtt | cgcagtctccaagaagctcta | 13 |
|  | *SIRT2* | Sirtuin 2 | ccatctgccactacttcatcc | ccactcgttccagagtgtca | 66 |
|  | *SIRT3* | Sirtuin 3 | cactacttccttcggctgct | ggcagggataccagatgct | 17 |
|  | *SIRT6* | Sirtuin 6 | ggacctaacgctcgctgat | ggtacccagggtgacagaca | 18 |
|  | *SNCA* | Synuclein Alpha | gctgctgctgagaaaacca | tccctccttagttttggaacc | 53 |
|  | *TAU (MAPT)* | Microtubule-associated protein tau | tggctccttggataacatca | aggtcagcttgtgggtttca | 25 |
