# Supplementary material for: β-Carbolines in Experiments on Laboratory Animals
Source: Int J Mol Sci. 2020 Jul 24;21(15):5245. doi: 10.3390/ijms21155245 (PMC7432475; doi:10.3390/ijms21155245)
Supplement: Supplementary file 1 [file ijms-21-05245-s001.zip › ijms-859032-supplementay/Supplementary Table S2.doc]

|  | **IDF [g]** | **SD** | **SDF [g]** | **SD** | **Protein [g]** | **SD** | **Fat [g]** | **SD** | **Ash [g]** | **SD** | **Water [%]** | **SD** |
| --- | --- | --- | --- | --- | --- | --- | --- | --- | --- | --- | --- | --- |
|  | **STAGE I** | | | | | | | | | | | |
| **C** | 54,52 | 2,16 | 0,07 | 0,01 | 14,94 | 0,59 | 0,76 | 0,28 | 81,63 | 1,35 | 16,10 | 4,60 |
| **H1** | 55,21 | 1,94 | 0,06 | 0,01 | 15,19 | 0,96 | 0,85 | 0,20 | 82,30 | 1,42 | 21,90 | 1,00 |
| **H2** | 56,86 | 0,33 | 0,06 | 0,01 | 16,45 | 1,31 | 1,16 | 0,20 | 82,32 | 0,41 | 17,40 | 3,00 |
| **H3** | 54,91 | 0,45 | 0,07 | 0,02 | 15,41 | 0,74 | 0,86 | 0,19 | 83,86 | 0,59 | 18,30 | 2,70 |
| **NH1** | 55,36 | 0,34 | 0,04 | 0,01 | 15,58 | 1,25 | 0,75 | 0,06 | 83,41 | 1,17 | 20,70 | 4,80 |
| **NH2** | 54,46 | 0,69 | 0,06 | 0,01 | 16,79 | 0,30 | 0,59 | 0,23 | 82,95 | 0,85 | 22,40 | 10,20 |
| **NH3** | 53,97 | 0,17 | 0,07 | 0,01 | 15,77 | 0,92 | 1,03 | 0,09 | 84,20 | 0,64 | 26,90 | 18,50 |
|  | **STAGE II** | | | | | | | | | | | |
| **C** | 55,69 | 1,87 | 0,04 | 0,03 | 15,39 | 1,02 | 0,81 | 0,38 | 82,39 | 0,20 | 15,30 | 2,80 |
| **CS** | 54,69 | 0,58 | 0,04 | 0,05 | 15,70 | 1,23 | 0,91 | 0,26 | 83,82 | 0,36 | 12,60 | 1,50 |

Supplementary Table 2. Mean content of fecal factors in 100g: insoluble dietary fiber (IDF), soluble dietary fiber (SDF), protein, fat, ash and water. Stage I and stage II.
